# Learning Disabilities in Reading and Writing and Type of Delivery in Twin Births

**DOI:** 10.3390/children8100834

**Published:** 2021-09-23

**Authors:** María-José González-Valenzuela, Dolores López-Montiel, Olga Cazorla-Granados, Ernesto-Santiago González-Mesa

**Affiliations:** 1 Department of Development and Educational Psychology, Faculty of Psychology, University of Málaga, Teatinos University Campus, s/n, 29071 Málaga, Spain; 2 Department of Psychobiology and Methodology of Behavioral Sciences, Faculty of Psychology, University of Málaga, Teatinos University Campus, s/n, 29071 Málaga, Spain; dlopezm@uma.es; 3 Santa Mª de la Victoria Teaching Foundation, Santa Rosa de Lima School, Argentea 19, 29006 Málaga, Spain; olgacazorla@gmail.com; 4 Department of Surgical Specialties, Biochemistry and Immunology, Faculty of Medicine, University of Málaga, Teatinos University Campus, s/n, 29071 Málaga, Spain; egonzalezmesa@gmail.com

**Keywords:** learning disabilities, reading, writing, type of delivery, twin births

## Abstract

The aim of this study was to analyse the relationship between the type of delivery (vaginal or caesarean), as a risk factor, and the likelihood of having learning disabilities in reading (reading accuracy) and writing (phonetic and visual orthography), controlling for the interaction and/or confounding effect of gestational, obstetric, and neonatal variables (maternal age at delivery, gestational age, foetal presentation, Apgar 1, and newborn weight) among six-year-old children born in twin births. In this retrospective cohort study, the exposed and non-exposed cohorts consisted of children born by caesarean section and vaginal delivery, respectively. A total of 124 children born in twin births were evaluated in year one of primary education. Intelligence was measured using the K-BIT test; reading and writing variables were evaluated using the Evalúa-1 battery of tests, and clinical records were used to measure gestational, obstetric, and neonatal variables. Binary logistic regressions applied to each dependent variable indicated that caesarean delivery is a possible independent risk factor for difficulties in reading accuracy and phonetic and visual orthography. Future research using larger samples of younger children is required to analyse the relationship between obstetric and neonatal variables and the different basic indicators of reading and writing.

## 1. Introduction

Twins have been considered in some studies as a psychologically and academically vulnerable population, even though this population is subject to the influence of certain prenatal and perinatal factors—hence, the interest of this study [1]. Multiple births are associated with prematurity, low Apgar scores, neonatal sepsis, pulmonary hypertension, hyperbilirubinaemia, and restricted intrauterine growth, among others. Some of these complications can on occasion lead to neuropsychological difficulties, academic difficulties, and even death [2,3,4,5,6].

Specific learning disabilities in reading and writing present disorders in cognitive processes and associative behaviours, which are explained by hypotheses on the biological origin thereof [7,8,9,10,11,12]. Pupils who display such difficulties have below the expected reading and writing level for their age, despite receiving adequate instruction for at least six months and having the intelligence to be a good reader [8,12]. They are characterised by difficulties in the precise and fluid recognition of words and by problems with spelling—in other words, difficulties with the basic components of reading and writing, such as reading and writing accuracy [7]. These specific difficulties affect lexical and sub-lexical processes (visual and phonological processing) implicit in both reading and writing accuracy, and they result from a deficit in the phonological component that is neurobiological in origin [7]. Estimates of the prevalence of learning disabilities vary by definition and language, but generally affect between 7% and 16% of school-age children [13,14]. There are studies that highlight the influence of pre- and perinatal factors in the appearance of cerebral dysfunctions, which would justify the existence of these difficulties in academic learning among children born in single and multiple births. Specifically, these factors could justify the characteristic cognitive and linguistic problems displayed [15,16,17,18], among others. Differences in the volume of grey matter in the cortex, reduced cortical activity, and disorders in cortical plasticity manifested by children with learning disabilities might be conditioned by the influence of these peri- and prenatal factors [19,20,21].

Prematurity would be one such perinatal risk factor [22,23,24,25,26,27,28,29,30,31]. Some of these studies find that children with low birth weight present reading difficulties, which vary according to weight gain and loss [24,25,30,31]. Other research states that children who weigh less than 1500 g at birth and are born before 34 weeks of gestation then go on to obtain lower scores than children born full term with regard to spelling, accuracy, and reading speed at young ages. These results are associated with the difficulties they display in terms of speech, phonological awareness, visual perception, rapid naming, and executive functions [23,26,27,32]. Furthermore, the *DSM-5* [7] indicates that one of the possible risk factors for specific learning disabilities is low birth weight.

Maternal age and foetal presentation are also considered risk factors in multiple births for psychological development and academic learning. Advanced maternal age increases the likelihood of multiple gestation [33], and extreme maternal age (teenagers and women over 35) has been described as an independent risk factor associated with adverse perinatal results [34]. Furthermore, the risk of complications such as preterm delivery is also higher in multiple pregnancies [35]. Foetal presentation conditions the type of delivery, with a high rate of caesarean sections when either of the foetuses is presenting non-cephalically. Vaginal delivery of the second twin is associated with lower scores on the Apgar test and lower umbilical cord pH in vaginal deliveries, depending on the interval between the births of both twins [36].

Another of the prenatal and obstetric factors that also seem to be related to academic learning and difficulties in twin births is type of delivery. From an obstetric point of view, there are no recommendations based on the analysis of prospective data in relation to the best delivery option for the second twin. In fact, current recommendations are based on retrospective studies, and the monitoring of children is based on the study of grave morbidity or mortality in the perinatal period [37]. One of the few prospective studies published about the influence of delivery type concludes that there are no differences in maternal or foetal morbidity in twins born vaginally or by caesarean section if they are both programmed and attended by qualified professionals, although the foetus born second does present a higher risk of morbidity than the first-born twin [38]. In other words, some studies indicate that there is no evidence that proves programmed caesarean delivery for twin births to be better than vaginal ones [3] and that it includes a risk of low Apgar scores, neonatal respiratory morbidity, perinatal mortality caused by the rupture of the uterus or by placenta previa, and placenta abruption in subsequent pregnancies [6,39,40]. However, other studies note that caesarean delivery reduces the risk of low scores in the Apgar 5 test in the first twin in breech presentation, foetal breech presentation, and intrapartum foetal death, but it increases the risk of both maternal and neonatal death in the event of cephalic presentation [3,6,41] and is associated with severe motor delay in early ages when performed under general anaesthesia [40]. Along these lines, previous studies have found that caesarean delivery is a risk factor for psychological development problems [41,42] and difficulties in reading (lexical access and comprehension), writing (phonetic and visual lexical access), and arithmetic in twin births [41,43]. However, these studies did not examine in depth the separate components of reading and writing, such as reading accuracy and phonetic and visual orthography. It would be interesting to analyse which of these components related to lexical and sub-lexical processes (visual and phonological processing) are influenced by the type of delivery since they are different cognitive processes.

Therefore, the main aim of this study of children born in twin births once they reached the age of six was to analyse the relationship between type of delivery (vaginal or caesarean), as a risk factor, and the probability of presenting learning disabilities in reading accuracy, phonetic orthography, and visual orthography, controlling for the interaction and/or confounding effect of other gestational, obstetric, and neonatal variables such as maternal age at delivery, gestational age, foetal presentation, Apgar 1 score, and newborn weight on account of their importance in neuropsychological development during childhood and specific academic learning.

## 2. Material and Methods

### 2.1. Design

To achieve the objective of this paper, the authors designed a retrospective epidemiological cohort study in which type of delivery (risk) predicted the learning of reading accuracy and visual and phonetic orthography among six-year-old children born in twin births (outcome). The exposed cohort comprised children born by caesarean section, and children from the sample selected who were born by natural or induced vaginal delivery comprised the non-exposed cohort

### 2.2. Participants

**Figure 1:**
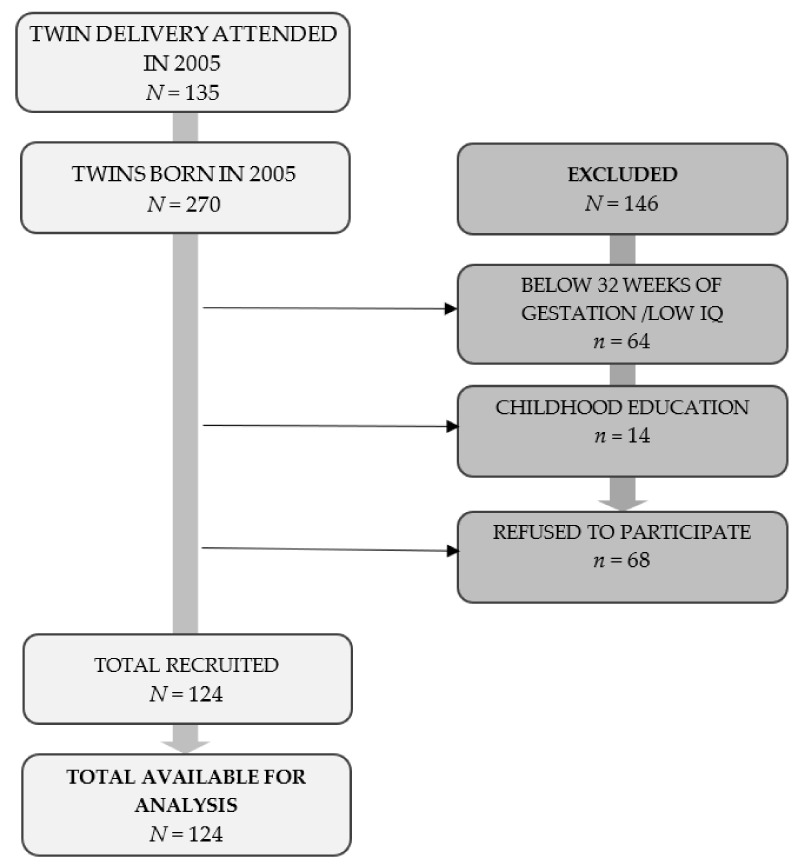
Flow diagram of participants through each stage of the study.

As in previous studies [41,42,43], the study population consisted of Caucasian children born in twin births at the Hospital Materno-Infantil deMálaga, once they had reached the age of six, who were born in 2005, and were in Year 1 of Primary Education. Of the 7120 births registered in the year 2005 in this hospital, 135 were twin births. Of the 270 children born of twin births that same year, 64 were excluded since they were born prior to 32 weeks of gestation, as were 14 who were still in Early Years Education and 68 who could not be recruited for the study owing to difficulties locating their families or because they did not wish to take part. Therefore, of the population selected, a total of 124 children could be evaluated, born in 62 twin births [41,42,43] (see Figure 1).

The age of the children was between 74 and 86 months (*M* = 79.42 and *SD* = 3.44), and the sample comprised 62 male (50%) and 62 female participants (50%) (Table 1).

The 124 children included in the final sample presented an average intelligence quotient (standard score = 101 and Enneatype type = 5). Of the 124 children included in the final sample, those with normal levels of intelligence (standard score ≥ 101 and E ≥ 5) who presented scores for reading and writing accuracy (phonetic and visual orthography) below the 25th percentile were classified as children with specific learning disabilities. The instruments used to evaluate intellectual quotient and reading and writing accuracy are defined in the instruments section. This criterion has been considered by different research studies to diagnose these subjects and coincides with the criterion established by the instrument used for the assessment of such disabilities [42,43,44,45,46,47]. In other words, performance in reading accuracy and phonetic and visual orthography was defined in terms of the following dichotomy: presence of difficulty in the learning of reading accuracy, phonetic orthography, or visual orthography, if the score achieved by the child in specific learning tests is below the 25th percentile; absence of difficulty in the learning of reading accuracy, phonetic orthography, or visual orthography, if the child achieves a grade of at least the 25th percentile in each measurement. A total of 93 children (75%) out of the total sample selected presented no difficulties in reading accuracy, 92 (74.2%) in phonetic orthography, and 93 (75%) in visual orthography. A total of 31 children (25%) presented difficulties in reading accuracy, 32 (25.8%) in phonetic orthography, and 31 (25%) in visual orthography (see Table 2, Table 3 and Table 4).

### 2.3. Instruments

To evaluate specific learning difficulties, different instruments were used.

To verify that the children did not have any intellectual disabilities, we measured their intelligence by means of the Kaufman Brief Intelligence Test (K-BIT) [48]. The K-BIT test analyses verbal and non-verbal intelligence from the age of four onwards and comprises two subtests: Vocabulary and Matrices. The first evaluated verbal abilities related to learning at school, using knowledge of words, and forming concepts (expressive vocabulary and definitions). The child had to say the name of a figure that was presented to him/her and find the word that best fit two clues that were given (a descriptive phrase and a word in which some letters had been deleted). The second test measured non-verbal abilities and the capacity to resolve problems, complete analogies, and perceive relations. It included two types of exercises consisting of 48 items: the first consisted of drawings of objects (e.g., a moon), and the child had to select from among 5 drawings that were placed (trousers, a sun, an apple, a car, and a heater) the one that best related to the stimulus drawing; the second demanded that the child complete a visual analogy from either figurative drawings or abstract figures. The total score for Intelligence was the sum total of the scores attained in each of the subtests (number of correct answers given). The reliability coefficients for the different tests ranged between 0.80 and 0.90 [48].

To analyse performance in the reading and writing measures applied here, we used different subtests from the Evalúa-1 psycho-pedagogical battery of tests [45]. The Evalúa-1 psycho-pedagogical battery is an instrument for children aged 6–7, which provides information about the cognitive foundations of learning, basic instrumental learning, and affective and behavioural aspects, in order to facilitate decision-making with regard to education processes. The three tests used encompassed reading accuracy, phonetic orthography, and visual orthography.

The Reading Accuracy test measured lexical access processes. These processes refer to knowledge of the main grapheme–phoneme conversion rules and fluency and pace in reading. Lexical access processes were measured by means of tasks to identify letters, syllables, words, pseudowords, and phrases. The child had to perform different tasks: to mark in each box the letter that was dictated to him (12 items), to match each letter from a column with that same letter from another column (14 items), to join with arrows each word from a column with the same word that was to its side (10 items), and to read aloud syllables, words and pseudowords (50 items), and phrases (2 items). The total score was obtained by adding together the number of correct answers given in the different tasks. The reliability of the test according to Cronbach’s alpha coefficient was 0.93 [45].

Phonetic orthography and visual orthography tests evaluate the phonetic and visual processes of lexical access which are involved in dictation, copying, and spontaneous writing. The phonetic orthography test measured knowledge of the phoneme–grapheme conversion rules through tasks involving the dictation of different linguistic units such as letters, syllables, words, and phrases; syllables, words, and phrases copying; and completing words in a short text. The sum total of correct answers given when carrying out the different tasks was the total score. The reliability of the test according to the Cronbach alpha coefficient was 0.97 [45]. The visual orthography test measured word recognition by means of a task involving completing graphemes that were missing everyday words with significant reference drawings. The total score was the sum total of correct answers given when carrying out the task. The reliability of the test according to the Cronbach alpha coefficient was 0.87 [45].

By analysing the clinical records of the mothers and their children during gestation and birth, we assessed gestational, obstetric, and neonatal variables. We then dichotomised the control variables analysed, in line with previous studies [41,42,43]: Maternal age at the time of delivery, over or under the age of 35; gestational age of the newborn, over or under 37 weeks; foetal presentation, cephalic or non-cephalic (breech or transverse); Minute-1 Apgar score, above or below seven points; and newborn weight, above or below 1500 grams.

### 2.4. Procedure

First, we requested authorisation from the Research Ethics Committee at the Hospital Materno Infantil (Comité Ético de Investigación-CEI), on 30 January 2014, in order to begin compiling data.

Second, we contacted the hospital administration directly to obtain the telephone details of mothers of twin births. Having contacted them, we explained how the research would be developed and then asked whether they would agree for their children to be subjected to psychological evaluation. At the beginning of each evaluation session, an informed consent form was signed by the mothers.

Third, the Kaufman Intelligence Test [48], and subsequently the reading and writing tests, were individually administered by three experienced psychologists. The approximate time taken was 40 min.

Finally, some of the authors involved in the study compiled the obstetric and neonatal data of the selected cases by reviewing the clinical records held at the hospital and through the identification of the mothers selected from all the records of twin births registered in the same year.

### 2.5. Statistical Analysis

According to the objective, design, and nature of the data, we chose regression models as the main statistical analysis procedure. To verify whether linear models were suitable for the data properties, we conducted descriptive and exploratory analyses of all the variables, performed a bivariate analysis, and estimated multiple linear regression models. For the bivariate analysis, Pearson’s correlation coefficients and biserial correlation coefficients were calculated according to the measurement scales of the variables and their corresponding significance tests. They were considered small (*r* = |0.10|), moderate (*r* = |0.30|), or strong (*r* = |0.50| or greater) according to Cohen’s criterion [49] for effect size. The independent variables were selected for each model when in the previous bivariate analyses they had an associated probability lower than 0.05 and an effect size equal to or greater than |0.20|. The non-fulfilment of the assumptions of linear regression (linearity, normality, and homoscedasticity) was verified both a priori and a posteriori. Therefore, in accordance with the properties of the data, the main statistical analysis technique chosen was binary logistical regression. To ensure the correct application of this technique, the dependent variable must be dichotomous categorical (measured on a nominal scale), whereas independent variables could be categorical or continuous. For continuous variables, the assumption of linearity between each continuous predictive variable and the logarithm of the response variable must be fulfilled. Having confirmed non-fulfilment, we included these dichotomised variables in the regression models.

Hence, to ensure efficient analysis and a clear interpretation of the results, we dichotomised all the variables that were originally continuous, both the dependent and the control variables, in accordance with the criteria set out previously, in line with previous studies [42,43].

Having explored all the variables, we applied different types of analyses to examine the study variables: preliminary analysis, binary logistic regression analysis, and complementary analysis to validate the regression models eventually estimated.

As part of the preliminary analyses, we carried out an initial descriptive analysis of the sociodemographic characteristics of the sample, the dependent variables, and the potential predictors analysed, in terms of means and standard deviations or in terms of frequencies and percentages, depending on the categorical or continuous nature of the variable according to its original scale. Subsequently, we compared the means or percentages of all the variables according to the type of delivery, with a view to evaluating the main relationships studied, detecting possible masking variables, and selecting the most appropriate ones for the regression models. We applied Student’s *t* tests for independent samples and Mann–Whitney U or Pearson’s χ2 tests depending on the nature of the variables, according to their original scale and the fulfilment of the assumptions for the application of parametric tests. We also studied the bivariate relationship between the study variables once they were dichotomised, by means of contingency tables and Pearson’s χ^2^ independence tests. For these preliminary analyses, we calculated the effect size of the statistics using Cohen’s *d*, the *r* correlation coefficient, and Cramer’s *V*, respectively [49,50]. Furthermore, to measure the degree to which type of delivery and each control variable increased or decreased the risk of having learning difficulties with regard to reading accuracy, phonetic orthography, and visual orthography, we estimated the unadjusted odds ratios (OR), along with their confidence intervals at 95% (95% CI).

In the exploratory analysis, the presence of outliers in the dependent variables was also analysed by means of the typified residuals and graphic analysis. When finding cases around two and three standard deviations above or below the mean, the logistic regression model was adjusted both with and without these cases. If there were no significant differences in the regression coefficients, in the ORs, and in the adjustment values between both models, the cases could be part of the sample.

In the binary logistic regressions for each dependent variable (difficulties with reading accuracy, phonetic orthography, and visual orthography), when statistically possible we assessed the possible interaction (modification of the principal effect studied) between the control variables and the independent variable type of delivery, as well as the possible confusion between the control variables and the main relationship evaluated (effect of the type of delivery on reading accuracy, phonetic orthography, and visual orthography). Variables were included when the previous bivariate analyses had more than 10% of cases for each cell in the contingency tables and a probability associated with Pearson’s χ^2^ statistic of less than 0.05.

The researchers [51,52] estimated the regression models based on a maximum hierarchical model, conserving statistically significant interactions and the variables involved, when possible. Having eliminated non-significant interactions sequentially from the model, we then went on to study possible confounding factors, examining the possible bias in the regression coefficients, the accuracy (amplitude) of their confidence intervals, and their standard error, as well as non-statistical criteria, such as change in the OR magnitude. This magnitude evaluated the strength of association between the independent and the dependent variable. The potential for confounding was observed when the magnitude of the OR clinically changed to a substantial degree (10% between the gross and adjusted measures of association) when eliminating one variable from the equation, with regard to the initial model. We also evaluated the effect size of the OR according to their transformation to Cohen’s *d* [53]. The variables retained were taken into account in the construction of the most suitable model. In order to evaluate the goodness of fit of the models, we used the Likelihood Ratio and Hosmer–Lemeshow tests. To compare the statistical significance of the regression coefficients, we applied Wald’s chi-squared test. For the global evaluation of the validity of the models, we used Nagelkerke’s adjusted coefficient of determination, along with the percentage of correct classifications. Since incorrect inferences can be drawn if correlations between observations with samples of twins are ignored, we validated the binary logistic regression models using a random sample selected from the total sample, comprising twins from different couples. Bearing in mind that, by halving the sample, statistical power is lost, we compared the results of these analyses with those obtained using bootstrapping techniques [54], with 1000 samples per analysis to simulate sampling and with robust estimations of standard errors, statistical significance, and confidence intervals for regression coefficients. All analyses were executed using version 23 of the Statistical Package for the Social Sciences (IBM SPSS).

## 3. Results

Table 1 provides the descriptive statistics for the variables from the study sample. A primary/pre-secondary level of education was shown by 51 mothers (41.1%) and 58 fathers (46.8%); an intermediate level of education (secondary or high school, technical and non-technical) was shown by 38 mothers (30.6%) and 40 fathers (32.3%); and 35 mothers (28.2%) and 26 fathers (21%) had a higher education (university and graduate). 

A total of 84 children were born vaginally (67.7%) and 40 via caesarean section (32.3%). An elected caesarean was indicated in 11 deliveries on account of maternal problems (preeclampsia, premature rupture of membranes, pregnancy following one or more caesarean births previously, abnormal contractions of the myometrium, or prolonged pushing), whereas in 29 deliveries, caesarean was indicated on account of foetal problems (first twin in wrong position, non-progression, or decline in foetal wellbeing). Foetal presentation was cephalic in 80 deliveries (64.5%) and non-cephalic (breech and transverse) in 44 deliveries (35.5%).

Maternal age at the time of delivery ranged from 22 to 45 years of age (*M* = 33.2 and *SD* = 4.29). Gestational age of the newborn was between 32 and 41 weeks (*M* = 35.14 and *SD* = 2.09). The score obtained in the 1-minute Apgar was between 4 and 10 points (*M* = 8.41 and *SD* = 1.18), and newborn weight was between 1179 and 3080 g (*M* = 2137.76 and *SD* = 432.79) 

Table 1 also provides descriptions of the variables from the study validation sample, showing that the characteristics of both samples are equivalent.

Table 2 summarises the description and comparisons between the means of the originally quantitative dependent variables (reading accuracy, phonetic orthography, and visual orthography) and the control variables (maternal age, gestational age, 1-minute Apgar, newborn weight, and foetal presentation); in accordance with the independent variable type of delivery (vaginal or caesarean), we observed statistically significant and moderate differences for reading accuracy (*U* = 1119.50, *z* = −2.99, *p* < 0.01, *r* = 0.26) between the mean rank of children born vaginally (*MR* = 69.17) and those born by caesarean section (*MR* = 48.49). The same was true for phonetic orthography (*U* = 1025.00, *z* = −3.50, *p* < 0.001, *r* = 0.31) between the mean ranks of the groups made up of the variable type of delivery, (*MR* = 70.30) vs. (*MR* = 46.13). Additionally, groups were different with respect to visual orthography (*U* = 1262.00, *z* = −2.24, *p* < 0.05, *r* = 0.20), (*MR* = 67.48) vs. (*MR* = 52.05).

With regard to the control variables, there were no statistically significant differences regarding maternal age (*U* = 1404.00, *z* = −1.48, *p* = 0.139, *r* = 0.13) between the mean rank of mothers who delivered by caesarean section (*MR* = 69.40) and those who delivered vaginally (*MR* = 59.21); for gestational age (*U* = 1664.00, *z* = −0.08, *p* = 0.932, *r* = 0.01) between the mean rank of children born vaginally (*MR* = 62.31) and those born by caesarean section (*MR* = 62.90); for Apgar 1 (*U* = 1586.00, *z* = −0.51, *p* = 0.612, *r* = 0.04) between the mean ranks of the two different groups, (*MR* = 61.11) vs. (*MR* = 63.85); or in newborn weight (*t* (122) = 0.62, *p* = 0.535, *d* = 0.12), as found by González-Valenzuela, González-Mesa et al. [42] and González-Valenzuela, López-Montiel et al. [43].

To detect possible interactions between the independent variable type of delivery (vaginal/caesarean) and the potentially masking control variables for the main effect (maternal age, gestational age, foetal presentation, newborn weight, and Apgar 1), we conducted bivariate analyses as seen in González-Valenzuela, González-Mesa et al. [42] and González-Valenzuela, López-Montiel et al. [43]. The only statistically significant relationship found was between type of delivery and foetal presentation (χ^2^(2, *N* = 124) = 45.53, *p* < 0.001, *V* = 0.60), with a large magnitude observed for this association (see Table 2).

To assess whether the type of delivery of one twin was related to the type of delivery of the other twin, a contingency table was also performed, considering the Pearson χ^2^ test of independence as well as the unadjusted OR and its 95% confidence interval. A total of 12 children (28.6%) were delivered vaginally after their brother was delivered by caesarean section, and another 12 children (60%) had a caesarean delivery after their brother had a vaginal delivery. On the other hand, 30 siblings were born both by vaginal delivery (71.4%) and 8 by caesarean section (40%). No statistically significant relationship was found between the type of birth of both children (χ^2^(1, *N* = 62) = 0.81, *p* = 0.368, *V* = 0.11). Therefore, the probability of being born by one type of delivery as a function of the other was not significant, OR = 1.66, 95% CI (0.54, 5.09).

In short, there were statistically significant differences observed related to the type of delivery in each one of the dependent variables (reading accuracy, phonetic orthography, and visual orthography). The mean values were significantly higher and with a medium effect size in children born vaginally compared with children born by caesarean section. For the control variables, the mean value was only significantly higher, with a large effect size, for the variable foetal presentation, where cephalic presentation was more frequent in vaginal deliveries, and non-cephalic delivery was more frequent in caesarean deliveries.

With the validation sample, the results of these preliminary analyses were also very similar to those of the study sample (see Table 2). The robust estimations of the statistics calculated by means of the 1000 sampling simulation samples also confirmed these results.

Table 3, Table 4 and Table 5, below, summarise the bivariate analyses between the independent variable, which is the type of delivery, and the control variables with each dependent variable (presence/absence of difficulty in reading accuracy, phonetic orthography, and visual orthography), respectively, having previously dichotomised the latter variables. In each table, the frequency distributions are presented for each level of the independent variables according to each of the dependent variables, the percentage of cells with expected frequencies below five, Pearson’s χ^2^ statistic, with its statistical significance and effect size, and the unadjusted OR and CI. ORs with intervals that did not contain the null value were considered significant (OR = 1). The percentage of cells with expected frequencies not less than five was only found in the relationship between newborn weight and each dependent variable.

As shown in Table 3, between the independent variable and the criterion variable and the control variables, the relationship between type of delivery and reading accuracy was statistically significant and moderate (χ^2^(2, *N* = 124) = 9.64, *p* < 0. 01, *V* = 0.28). Of the 40 (32.3%) children born by caesarean section, 17 (42.5%) did not pass the reading accuracy test, whereas of the 84 (67.7%) born by vaginal delivery, 14 (16.7%) did not pass. We estimated the OR to evaluate the frequency of difficulties in learning reading accuracy present in children who were born by caesarean section in comparison with children born vaginally. This raw measure seems to indicate that birth by caesarean triples the likelihood of presenting difficulties in reading accuracy, OR = 3.69, 95% CI (1.58, 8.64). We did not observe any statistically significant relationships between reading accuracy and the other control variables evaluated.

Table 4 shows that the main relationship between type of delivery and phonetic orthography was also statistically significant and the magnitude of the relationship moderate (χ^2^(2, *N* = 124) = 8.59, *p* < 0. 01, *V* = 0.26). Of the 40 (32.3%) children born by caesarean section, 17 (42.5%) did not pass the phonetic orthography test, and of the 84 (67.7%) children born vaginally, 15 (17.9%) did not pass. The OR seems to indicate that caesarean delivery triples the likelihood of presenting difficulties in this dependent variable, OR = 3.40, 95% CI (1.47, 7.87) (see Table 3). The same was true between foetal presentation and phonetic orthography (χ^2^(2, *N* = 124) = 8.12, *p* < 0.01, *V* = 0.25). Of the 44 (35.5%) children born following non-cephalic presentation, 18 (40.9%) did not succeeded phonetic orthography test, and 14 (17.5 %) out of the 80 (64.5%) born following cephalic presentation did not pass. The OR was statistically significant, OR = 3.26, 95% CI (1.42, 7.51), indicating once again a likelihood of presenting difficulties with regard to phonetic orthography approximately 3 times greater among children who were born following non-cephalic presentation.

Table 5 also shows a significant and moderate relationship between type of delivery and visual orthography (χ^2^(2, *N* = 124) = 7.08, *p* < 0.01, *V* = 0.24). Of the 40 (32.3%) children born by caesarean section, 16 (40%) did not pass the visual orthography test, and of the 84 (67.7%) born vaginally, 15 (17.9%) did not pass. In this case, the probability of presenting difficulties in visual orthography is approximately 3 times higher when the type of delivery was by caesarean section rather than vaginal, OR = 3.06, 95% CI (1.32, 7.13). We did not find any statistically significant relationships between visual orthography and the other control variables studied here. We observed similar results in the validation sample with regard to these bivariate analyses. All the relationships were statistically significant and moderate between type of delivery and difficulties with reading accuracy (χ^2^(1, *N* = 62) = 4.58, *p* < 0.05, *V* = 0.27), type of delivery and difficulties with phonetic orthography (χ^2^(1, *N* = 62) = 5.20, *p* < 0.05, *V* = 0.29), foetal presentation and difficulties with phonetic orthography (χ^2^(1, *N* = 62) = 7.06, *p* < 0.01, *V* = 0.34), as well as between type of delivery and difficulties with visual orthography (χ^2^(1, *N* = 62) = 7.56, *p* < 0.05, *V* = 0.35). However, on account of the sample size, in the distribution of frequencies, percentages of cells with expected frequencies no lower than five were found in the relationship between maternal age, newborn weight, and 1-minute Apgar and each dependent variable. The unadjusted ORs were also statistically significant, with a moderate effect size, according to their CI (see the ORs calculated in the univariate logistic regression analysis shown in Table 6).

We subsequently carried out binary logistic regressions for reading accuracy, phonetic orthography, and visual orthography according to the type of delivery to evaluate the degree to which this factor increased or decreased the risk of having difficulties in these aptitudes, statistically controlling for possible interactions and confounding factors. The results are summarised in Table 6.

With the independent variable difficulties in reading accuracy, the estimated model was significant (χ^2^(1, *N* = 124) = 9.21, *p* < 0.01), including only the independent, with an OR = 3.69, 95% CI (1.58, 8.64). In the model presented, birth by caesarean section presented a risk factor for difficulties in reading accuracy: the risk was 3.69 times higher among those born by caesarean sector than those born vaginally in twin births once these children have reached the age of 6. The magnitude of the effect of this odds ratio was moderate, according to the transformation into Cohen’s *d*. Regarding the explanatory capacity of this model, 10% of the variability observed in the response variable was explained by the estimated logistic regression model (Nagelkerke R^2^ = 0.10). The percentage of cases that could be correctly predicted was 75% (see Table 6).

Secondly, we adjusted the main relationship studied between type of delivery and difficulties in phonetic orthography for foetal presentation and interaction between type of delivery and foetal presentation. Since foetal presentation was related statistically to the independent variable (χ^2^(2, *N* = 124) = 45.53, *p* < 0.001) and the dependent variable (see Table 4), it was considered a potential modifying variable of the main effect studied, as well as a potential confounding variable. According to this adjustment, having eliminated in two successive steps the interaction term and the variable foetal presentation, the third estimated model was statistically significant (χ^2^(1, *N* = 124) = 8.23, *p* <0.01), where the independent variable was significant with an OR = 3.40, 95% CI (1.47, 7.87). Model 2 shows that the effect of the variable type of delivery on difficulties in phonetic orthography was modified in the presence of the variable foetal presentation, but the ORs were not statistically significant (they included unity), and so it was not included in the final model. Caesarean birth was a risk factor for difficulties in learning phonetic orthography, making the risk 3.40 times higher among those born by caesarean section than the children born through vaginal delivery in twin births, once the children reached the age of 6. According to the OR, the effect size was considered small. In this model, the estimated model explained 9% of variance in the variable reading accuracy (Nagelkerke R^2^ = 0.09). The percentage of cases it could correctly predict was 74.2% (see Table 6). 

With the dependent variable visual orthography, two cases were detected close to 3 standard deviations below the mean. A posteriori, the regression model for visual orthography was adjusted both with these two cases and without them, not appreciating significant differences in the regression coefficients, in the OR, or in the adjustment values of the models between both procedures. Therefore, it was decided that these two cases could be part of the sample.

The estimated model was significant (χ^2^(1, *N* = 124) = 6.79, *p* < 0.01), including only the independent variable type of delivery, with an OR = 3.06 and 95% CI (1.32, 7.13). Therefore, the risk of having difficulties in the learning of visual orthography was 3.06 times higher among those born by caesarean than those children born via vaginal delivery in twin births, once the children reached the age of 6. According to the OR, the effect size was also considered small. In the estimated model, the variable type of delivery explained 8% of the variance in the variable visual orthography (Nagelkerke R^2^ = 0.08). The percentage of cases correctly predicted was 75% (see Table 6).

With the random sample of 62 subjects, comprising one twin from each couple, we found similar results to those obtained with the total sample, for all the variables studied (see Table 6).

In summary, the differences between the mean values show significantly higher scores in the learning variables for children born vaginally. In bivariate associations, type of delivery had a significant effect on the probability of presenting difficulties in the learning of reading accuracy, phonetic orthography, and visual orthography. With regard to the other gestational, obstetric, and neonatal variables, only foetal presentation appeared as a potential modifying and confounding variable for the main effect between type of delivery and phonetic orthography. However, although related to both, when it was controlled statistically by means of logistic regression, the effect of the first on the second was not modified. Therefore, in line with the preliminary statistical analysis, only the variable type of delivery was shown as a possible risk factor for disabilities in the reading and writing measures taken into account in this study.

## 4. Discussion

The objective of this cohort study of children born in twin births once they reached the age of 6 was to analyse retrospectively the relationship between reading accuracy and phonetic and visual orthography with type of delivery, evaluating the degree to which type of delivery may be related to the risk of having difficulties in these basic and specific components of reading and writing, and controlling statistically for possible interacting and confounding factors.

We observed that type of delivery was related statistically to the learning disabilities found, constituting a risk factor. The risk of having difficulties in reading accuracy and phonetic and visual orthography can be around 3 times higher among the children born by caesarean section than those born through vaginal delivery. The magnitude of the effect of type of delivery was moderate in relation to reading accuracy, and small in relation to phonetic and visual orthography. The explanatory capacity of variance in reading and writing learning disabilities was discreet, as was the percentage of cases that the final models could predict adequately, in accordance with final models that only included one independent variable.

In other words, in multiple births where caesarean deliveries must be performed, there are certain obstetric and perinatal circumstances that might affect the neurological development of the baby [17]. These types of conditions would justify in the long term the linguistic and cognitive difficulties that characterise the difficulties in reading accuracy and in phonetic and visual orthography. Recent studies have found that type of delivery is related to and is also a risk factor for neuropsychological development disorders and intellectual alterations in twin births [41,42,43]. Furthermore, some studies highlight the existence of short-term neonatal morbidity related with caesarean delivery, describing high rates of neonatal hypoxia, transient tachypnoea, and respiratory distress syndrome in children born by caesarean section [38], with a potential influence on posterior neurocognitive development. Various studies also highlight an increase in long-term postnatal morbidity, with an increase in respiratory morbidity, such as asthma or obstructive apnoea, diabetes, and obesity [3,6,41]. Our results also support the existence of circumstances that unfavourably condition the development of children born by caesarean section and justify the cognitive and linguistic difficulties they present [3,6,19,41,42,43,44,45,55]. Although the physio-pathological mechanisms underlying the deficits described are not clear, it would appear that the most striking difference between children born vaginally and those born via a programmed caesarean section is the neuroendocrine response to the stress produced by contractions, conditioned by normal delivery [6,56]. These differences in the neuroendocrine response to stress have been linked, in the case of programmed caesarean births, with the existence of defective expressions of certain genes (UCP2) in the neurons on the foetal hippocampus [55], with differences in the concentrations of dopamine depending on the type of delivery in certain areas of the prefrontal cortex, the nucleus accumbens, and striatum [57,58] and with differences in concentrations of noradrenaline in the adult amygdala and thalamus [59].

It should be noted that, in our study, gestational age and newborn weight did not affect reading and writing variables, as other studies have found [42,45]. This might be due to the fact that the choice of the sample excluded extremely premature or very premature subjects, whose psychological development and academic performance might truly be affected [22,23,25,27,30].

In short, although in multiple births caesarean section delivery is a risk factor for neuropsychological development disorders and specific learning difficulties [41,42,43,44,45], the results found in this study also indicate risk in the basic components of reading and writing related to separate lexical and sub-lexical processes, such as reading accuracy and phonetic and visual orthography. These results could have important repercussions in the explanation of dyslexia and are in line with the findings put forward by some studies about the influence of perinatal factors on school learning [19,20,41,42,43,44,45]. However, they should be taken with caution since they do not take into account other potentially influential obstetric and perinatal variables. 

Future studies should be conducted using broader samples in order to adequately control for variables, such as newborn weight, so that the findings can be generalized.. Furthermore, in order to analyse which other types of explanations could lead to the appearance of specific components of reading and writing difficulties, it would also be advisable to include other obstetric and perinatal variables as possible risk factors (e.g., congenital infection, antenatal drug/toxin exposure, respiratory distress, hyperbilirubinaemia, etc.). Some research has found that the risk of exhibiting reading and spelling problems among children with normal intelligence levels is increased when there is perinatal asphyxia [60] and that neonatal hyperbilirubinaemia is a risk factor for problems with reading, writing, and mathematics [61]. Along this line, a maternal exposure to nicotine during pregnancy is related with the DYX1C1 gene and would justify problems decoding words in reading and writing [8,62,63]. The role played by the reason for caesarean section should also be taken into consideration—and this is a limitation of the present study—since foetal stress caused by possible infection, deficient blood flow, etc., is probably what puts children at greater risk of developing learning disabilities, although the decision to opt for a caesarean is made the moment the attending physicians observe that this situation might occur, using their clinical judgement. Knowing the influence of sociodemographic variables (parents’ level of education and profession, etc.) and their interaction with the aforementioned perinatal and obstetric variables would also be useful in terms of analysing which other types of explanations might give rise to the appearance of specific difficulties in reading and writing. 

Furthermore, the findings of this study could be useful in clinical practice since they support the avoidance of caesarean section on demand or without specialised indication, in order to avoid in the long term the appearance of specific difficulties in reading (reading accuracy) and writing (phonetic and visual orthography). They also point to the advantages of vaginal delivery in multiple pregnancies, provided it is not contraindicated.

In conclusion, this study opens up new possibilities for research since the type of delivery has consequences in the learning of reading and writing among students born in twin births. Although, according to the results, clinical relevance is not high, it is also not insignificant and should not be ignored. Many factors are involved in the choice of delivery in twin births, and these must be studied (reason for caesarean, congenital infection, hyperbilirubinaemia, respiratory distress, etc.). Therefore, further research is needed with larger samples that will provide more accurate information about the relevance of such factors.

## Figures and Tables

**Table 1:** Characteristics of the samples.

Variables	*N* = 124 (Study Sample)			*N* = 62 (Validation Sample)		
	*n* (%)	*M (SD)*	Range	*n* (%)	*M (SD)*	Range
**Age (months)**		79.42 (3.44)	74–86		79.42 (3.46)	74–86
**Gender**						
Female	62 (50)			34 (50)		
Male	62 (50)			28 (45.2)		
**Mother’s level of education**						
Low	51 (41.1)			25 (40.3)		
Middle	38 (30.6)			19 (30.6)		
High	35 (28.2)			18 (29)		
**Father’s level of education**						
Low	58 (46.8)			29 (46.8)		
Middle	40 (32.3)			20 (32.3)		
High	26 (21)			13 (21)		
**Type of delivery**						
Vaginal	84 (67.7)			42 (67.7)		
Caesarean	40 (32.3)			20 (32.3)		
**Foetal presentation**						
Cephalic	80 (64.5%)			41 (66.1%)		
Non-cephalic	44 (35.5%)			21 (33.9%)		
**Maternal Age (years)**		33.24 (4.29)	22–45		33.24 (4.29)	22–45
**Gestational Age (weeks)**		35.14 (2.09)	32–41		35.14 (2.09)	32–41
**Weight of Newborn (grams)**		2137.76 (432.79)	1179–3080		2170.90 (433.66)	1250–3080
**Apgar 1**		8.41 (1.18)	4–10		8.36 (1.25)	5–9
**Reading Accuracy**		110.18 (27.21)	25–144		107.82 (29.25)	25–144
**Phonetic Orthography**		61.60 (12.67)	11–78		60.29 (15.31)	11–78
**Visual Orthography**		16.12 (6.26)	0–28		16.23 (6.36)	0–26

*Note*. *M* = mean; *SD* = standard deviation; Low: primary and pre-secondary studies; Middle: secondary or high school (technical and non-technical); High: university and graduate.

**Table 2:** Characteristics of the samples for both vaginal delivery and caesarean delivery.

Variables	Type of Delivery													
	Vaginal Delivery					Caesarean								
***N*** **= 124** **Study Sample**	***n*** **= 84 (66.7%)**					***n* = 40 (32.3%)**					**Statistical Test**			
	***n*** **(%)**	***M***	***SD***	**Range**	**MR**	***n*** **(%)**	***M***	***SD***	**Range**	**MR**	***U***	***Z***	***p***	**ES**
Reading Accuracy		114.73	25.35	26–144	69.17		100.63	28.81	25–144	48.49	1119.50	−2.99	0.003	0.26 ^1^
Phonetic Orthography		64.06	11.68	11–78	70.30		56.43	13.26	25–78	46.13	1025.00	−3.50	0.000	0.31 ^1^
Visual Orthography		17.06	5.46	0–26	67.48		14.15	7.36	0–28	52.05	1262.00	−2.24	0.025	0.20 ^1^
														
Maternal Age (years)		32.71	4.03	22–40	59.21		34.35	4.42	28–45	69.40	1404.00	−1.48	0.139	0.13 ^1^
Gestational Age (weeks)		35.09	1.99	32–40	62.31		35.25	2.27	32–41	62.90	1664.00	−0.08	0.932	0.01 ^1^
Apgar 1		8.36	1.23	4–10	61.11		8.53	1.06	5–9	63.85	1586.00	−0.51	0.612	0.04 ^1^
											*t*	*df*		
Weight of Newborn (grams)		2154.48	445.26	1310–3080	−		2102.65	408.59	1179–2905	−	0.62	122	0.535	0.12 ^2^
Foetal presentation											χ^2^			
Cephalic	71 (84.5)					9 (22.5)					45.53	1	0.000	0.60 ^3^
Non-cephalic	13 (15.5)					31 (77.5)								
***N*** **= 62** **Validation Sample**	***n*** **= 42 (66.7%)**					***n*** **= 20 (32.3%)**								
	***n*** **(%)**	***M***	***SD***	**Range**	**MR**	***n*** **(%)**	***M***	***SD***	**Range**	**MR**	***U***	***Z***	***p***	**ES**
Reading Accuracy		112.69	27.88	26–143	35.11		97.60	30.13	25–144	23.93	268.50	−2.28	0.022	0.29 ^1^
Phonetic Orthography		64.05	14.56	11–78	36.96		52.40	14.07	25–76	20.03	190.50.	−3.46	0.001	0.44 ^1^
Visual Orthography		17.48	5.19	3–26	34.74		13.60	7.81	0–25	24.70	284.00	−2.05	0.040	0.26 ^1^
														
Maternal Age (years)		32.81	3.20	22–40	30.31		34.15	4.66	28–45	34.00	370.00	−0.75	0.450	0.09 ^1^
Gestational Age (weeks)		35.00	2.03	32–40	30.43		35.44	2.22	32–41	33.75	375.00	−0.68	0.497	0.08 ^1^
Apgar 1		8.37	1.22	5–9	31.07		8.53	1.35	5–9	30.85	407.00	−0.06	0.951	0.01 ^1^
											*t*	*df*		
Weight of Newborn (grams)		2140.93	456.88	1310–3080	−		2233.85	383.65	1250–2905	−	−0.78	60	0.435	0.22 ^2^
Foetal presentation		112.69	27.88	26–143	35.11		97.60	30.13	25–144	23.93	χ^2^			
Cephalic	37 (88.1)					4 (20)					28.05	1	.000	0.67 ^3^
Non-cephalic	5 (11.9)					16 (80)								

*Note*. *M* = mean; *SD* = standard deviation*;* MR = rean rank; *U =* Mann–Whitney U-test; *t* = Student’s *t*-test; Pearson χ^2^; ES = effect size. ^1^ Correlation coefficient *r* (Cohen’s reference values: small = 0.10; medium = 0.30; large = 0.50; very large = 0.70). ^2^ Cohen’s *d* (Cohen’s reference values: small = 0.20; medium = 0.50; large = 0.80). ^3^ Cramer’s *V* (Cramer’s *V* reference values for *df less* = 1: small = 0.10; medium = 0.30; large = 0.50).

**Table 3:** Contingency tables and bivariate associations between reading accuracy, type of delivery, and the control variables.

Variables	Categories	Total *N* = 124	Dependent Variable Reading Accuracy		Test ^1^	Sig.	ES ^2^	OR	95% CI	
			No RAD *n* = 93 (75%)	RAD *n* = 31 (25%)					Lower	Upper
**Independent** **Type of delivery**	Vaginal Caesarean	84 (67.7%) 40 (32.3%)	70 (83.3%) 23 (57.5%)	14 (16.7%) 17 (42.5%)	9.64 ^a^	0.002	0.28	3.69	1.58	8.64
**Control** Maternal age (years)	Under 35 Over 35	88 (71%) 36 (29%)	66 (75%) 27 (75%)	22 (25%) 9 (25%)	0.00 ^a^	0.999	0.00	1.00	0.41	2.45
Gestational age of newborn (weeks)	Over 37 Under 37	40 (32.3%) 84 (67.7%)	29 (72.5%) 64 (76.2%)	11 (27.5%) 20 (23.8%)	0.19 ^a^	0.657	0.04	0.82	0.35	1.94
Foetal presentation	Cephalic Non-cephalic	80 (64.5%) 44 (35.5%)	62 (77.5%) 31 (70.5%)	18 (22.5%) 13 (29.5%)	0.75 ^a^	0.386	0.08	1.44	0.63	3.32
Weight of newborn (grams)	Over 1500 Under 1500	112 (90.3%) 12 (9.7%)	83 (74.1%) 10 (83.3%)	29 (25.9%) 2 (16.7%)	0.49 ^b^	0.729	0.06	0.57	0.12	2.77
Apgar 1	Over 7 Under 7	99 (79.8%) 25 (20.2%)	75 (75.8%) 18 (72%)	24 (24.2%) 7 (28%)	0.15 ^a^	0.698	0.03	1.21	0.45	3.26

*Note*. RAD = Reading Accuracy Difficulty; ES = effect size; OR = odds ratio; CI = confidence interval. ^1^ Pearson’s χ^2^. ^2^ Cramer’s *V* (Cramer’s *V* reference values for *df less* = 1: small = 0.10; medium = 0.30; large = 0.50). ^a^ 0% of cells with an expected frequency less than 5. ^b^ 25% of cells with an expected frequency less than 5.

**Table 4:** Contingency tables and bivariate associations between phonetic orthography, type of delivery, and the control variables.

Variables	Categories	Total *N* = 124	Dependent Variable Phonetic Orthography		Test ^1^	Sig.	ES ^2^	OR	95% CI	
			No POD *n* = 92 (74.2%)	POD *n* = 32 (25.8%)					Lower	Upper
**Independent** **Type of delivery**	Vaginal Caesarean	84 (67.7%) 40 (32.3%)	69 (82.1%) 23 (57.5%)	15 (17.9%) 17 (42.5%)	8.59 ^a^	0.003	0.26	3.40	1.47	7.87
**Control** Maternal age (years)	Under 35 Over 35	88 (71%) 36 (29%)	69 (78.4%) 23 (63.9%)	19 (21.16%) 13 (36.1%)	2.81 ^a^	0.093	0.15	2.05	0.88	4.79
Gestational age ofnewborn (weeks)	Over 37 Under 37	40 (32.3%) 84 (67.7%)	31 (77.5%) 61 (72.6%)	9 (22.5%) 23 (27.4%)	0.33 ^a^	0.561	0.05	1.30	0.53	3.14
Foetal presentation	Cephalic Non-cephalic	80 (64.5%) 44 (35.5%)	66 (82.5%) 26 (59.1%)	14 (17.5%) 18 (40.9%)	8.12 ^a^	0.004	0.25	3.26	1.42	7.51
Weight of newborn (grams)	Over 1500 Under 1500	112 (90.3%) 12 (9.7%)	82 (73.2%) 10 (83.3%)	30 (26.8%) 2 (16.7%)	0.58 ^b^	0.729	0.07	0.54	0.11	2.64
Apgar 1	Over 7 Under 7	99 (79.8%) 25 (20.2%)	75 (75.8%) 17 (68%)	24 (24.2%) 8 (32%)	0.63 ^a^	0.478	0.07	1.47	0.56	3.83

*Note*. POD = Phonetic Orthography Difficulty; ES = effect size; OR = odds ratio; CI = confidence interval. ^1^ Pearson’s χ^2^. ^2^ Cramer’s *V* (Cramer’s *V* reference values for *df less* = 1: small = 0.10; medium = 0.30; large = 0.50). ^a^ 0% of cells with an expected frequency less than 5. ^b^ 25% of cells with an expected frequency less than 5.

**Table 5:** Contingency tables and bivariate associations between visual orthography, type of delivery and the control variables.

Variables	Categories	Total *N* = 124	Dependent Variable Visual orthography		Test ^1^	Sig.	ES ^2^	OR	95% CI	
			No VOD *n* = 93 (75%)	VOD *n* = 31 (25%)					Lower	Upper
**Independent** **Type of delivery**	Vaginal Caesarean	84 (67.7%) 40 (32.3%)	69 (82.1%) 24 (60%)	15 (17.9%) 16 (40%)	7.08^a^	0.008	0.24	3.06	1.32	7.13
**Control** Maternal age (years)	Under 35 Over 35	88 (71%) 36 (29%)	67 (76.1%) 26 (72.2%)	21 (23.9%) 10 (27.8%)	0.21 ^a^	0.648	0.04	1.23	0.51	2.95
Gestational age of newborn (weeks)	Over 37 Under 37	40 (32.3%) 84 (67.7%)	29 (72.5%) 64 (76.2%)	11 (27.5%) 20 (23.8%)	0.19 ^a^	0.657	0.04	0.82	0.35	1.94
Foetal presentation	Cephalic Non-cephalic	80 (64.5%) 44 (35.5%)	64 (80%) 29 (65.9%)	16 (20%) 15 (34.1%)	3.00 ^a^	0.083	0.15	2.07	0.90	4.74
Weight of newborn (grams)	Over 1500 Under 1500	112 (90.3%) 12 (9.7%)	85 (75.9%) 8 (66.7%)	27 (24.1%) 4 (33.3%)	0.49 ^b^	0.493	0.06	1.57	0.44	5.64
Apgar 1	Over 7 Under 7	99 (79.8%) 25 (20.2%)	77 (77.8%) 16 (64%)	22 (22.2%) 9 (36%)	2.00 ^a^	0.155	0.13	1.97	0.76	5.06

*Note*. VOD = Visual Orthography Difficulty; ES = effect size; OR = odds ratio; CI = confidence interval. ^1^ Pearson’s χ^2^. ^2^ Cramer’s *V* (Cramer’s *V* reference values for *df less* = 1: small = 0.10; medium = 0.30; large = 0.50). ^a^ 0% of cells with an expected frequency less than 5.

**Table 6:** Multivariate logistic regression analysis for reading accuracy and phonetic and visual orthography disabilities, adjusted by potential interaction and confounding factors.

***N* = 124**	***Variables***	***b***	***SE***	***Wald* χ^2^**	***df***	***p***	**OR**	**95% CI**	
**Study Sample**								***Lower***	***Upper***
**RAD**	**Type of delivery ^(a)^**	1.31	0.43	9.08	1	0.003	3.69 ^1^	1.58	8.64
Model 1	Constant	−1.61	0.29	30.22	1	0.000	0.20		
* χ^2^(1, *N* = 124) = 9.21, *p* = 0.002; R^2^ = 0.10; GPC = 75%									
**POD**	Type of delivery ^(a)^	1.00	0.77	1.65	1	0.198	2.72	0.59	12.56
Model 1	Foetal presentation ^(b)^	0.88	0.68	1.67	1	0.196	2.42	0.63	9.27
	Type of delivery x Foetal presentation	−0.38	1.05	0.13	1	0.712	0.68	0.08	5.30
	Constant	−1.69	0.33	26.75	1	0.000	0.18		
* χ^2^(3, *N* = 124) = 10.20, *p* = 0.017; χ^2^(3, *N* = 124) = 0.00, *p* = 0.999; R^2^ = 0.11; GPC = 74.2%									
Model 2	Type of delivery ^(a)^	0.79	0.53	2.19	1	0.139	2.20	0.77	6.25
	Foetal presentation ^(b)^	0.72	0.53	1.85	1	0.173	2.05	0.73	5.81
	Constant	−1.66	0.31	28.90	1	0.000	0.19		
* χ^2^(2, *N* = 124) = 10.06, *p* = 0.007; χ^2^(2, *N* = 124) = 0.13, *p* = 0.934; R^2^ = 0.11; GPC = 74.2%									
Model 3	**Type of delivery** ^(a)^	1.22	0.43	8.16	1	0.004	3.40 ^1^	1.47	7.87
	Constant	−1.52	0.28	28.69	1	0.000	0.21		
* χ^2^(1, *N* = 124) = 8.23, *p* = 0.004; R^2^ = 0.09; GPC = 74.2%									
**VOD**	**Type of delivery** ^(a)^	1.12	0.43	6.77	1	0.009	3.06 ^1^	1.32	7.13
Model 1	Constant	−1.52	0.28	28.69	1	0.000	0.21		
* χ^2^(1, *N* = 124) = 6.79, *p* = 0.009; R^2^ = 0.08; GPC = 75%									
***N*** **= 62**	***Variables***	***b***	***SE***	***Wald*** **χ** **^2^**	***df***	***p***	**OR**	**95% CI**	
Validation Sample								***Lower***	***Upper***
**RAD**	**Type of delivery** ^(a)^	1.24	0.60	4.36	1	0.037	3.48 ^1^	1.08	11.20
Model 1	Constant	−1.45	0.39	13.56	1	0.000	0.23		
* χ^2^(1, *N* = 62) = 4.41, *p* = 0.036; R^2^ = 0.10; GPC = 72.6%									
**POD**	Type of delivery ^(a)^	0.36	1.23	0.08	1	0.772	1.43	0.13	15.87
Model 1	Foetal presentation ^(b)^	1.05	1.00	1.09	1	0.296	2.86	0.40	20.47
	Type of delivery x Foetal presentation	0.30	1.61	0.03	1	0.852	1.35	0.06	31.77
	Constant	−1.69	0.33	26.75	1	0.000	0.18		
* χ^2^(3, *N* = 62) = 7.36, *p* = 0.006; χ^2^(3, *N* = 62) = 0.00, *p* = 0.999; R^2^ = 0.16; GPC = 72.6%									
Model 2	Type of delivery ^(a)^	0.53	0.78	0.46	1	0.498	1.70	0.37	7.85
	Foetal presentation ^(b)^	1.17	0.77	2.28	1	0.131	3.21	0.70	14.67
	Constant	−1.47	0.41	13.15	1	0.000	0.23		
* χ^2^(2, *N* = 62) = 7.32, *p* = 0.026; χ^2^(2, *N* = 62) = 0.03, *p* = 0.983; R^2^ = 0.16; GPC = 72.6%									
Model 3	**Type of delivery** ^(a)^	1.30	0.58	4.94	1	0.026	3.67 ^1^	1.17	11.52
	Constant	−1.30	0.37	11.94	1	0.001	0.27		
* χ^2^(1, *N* = 62) = 5.04, *p* = 0.025; R^2^ = 0.11; GPC = 69.4%									
**VOD**	**Type of delivery** ^(a)^	1.61	0.61	6.97	1	0.008	5.00^1^	1.51	16.51
Model 1	Constant	−1.61	0.41	15.11	1	0.000	0.20		
* χ^2^(1, *N* = 62) = 7.26, *p* = 0.007; R^2^ = 0.16; GPC = 72.6%									

*Note.* RAD = Reading Accuracy Difficulty; POD = Phonetic Orthography Difficulty; VOD = Visual Orthography Difficulty; OR = odds ratio; CI = confidence interval. Variables reference categories: ^(a)^ = Vaginal delivery; ^(b)^ = Cephalic. ^1^ OR effect size as a function of the transformation to Cohen’s *d* (Cohen’s reference values: insignificant = less than 1.68; small = 1.68–3.47; moderate = 3.47–6.71; large = greater than 6.71). * Goodness-of-fit tests for logistic regression models: global test χ^2^; Hosmer–Lemeshow χ^2^; Nagelkerke R^2^; GPC = global percentage of correct classifications.

## Data Availability

No data availability. This manuscript does not have associated data in a data repository.

## References

[1] Allin A.B., Fischbein S. (1991). Twins: Are they at risk? A longitudinal study of twins and nontwins from birth to 18 years of age. Acta Genet. Med. Gemellol..

[2] Carlsson Wallin M., Ekström P., Marsál K., Källé K. (2010). Apgar score and perinatal death after one previous caesarean delivery. BJOG.

[3] Hogle K.L., Hutton E.K., McBrien K.A., Barrett J.F., Hannah M.E. (2003). Cesarean delivery for twins: A systematic review and meta-analysis. Am. J. Obstet. Gynecol..

[4] Liston F.A., Allen V.M., O’Connell C.M., Jangaard K.A. (2008). Neonatal outcomes with caesarean delivery at term. Arch. Dis. Child Fetal Neonatal Ed..

[5] Olusanya B.O. (2011). Perinatal outcomes of multiple births in southwest Nigeria. J. Health Popul. Nutr..

[6] Villar J., Carroli G., Zavaleta N., Donner A., Wojdyla D., Faundes A., Velazco A., Bataglia V., Langer A., Narváez A. (2007). Maternal and neonatal individual risks and benefits associated with caesarean delivery: Multicentre prospective study. BMJ.

[7] American Psychiatric Association (APA) (2014). Diagnostic and Statistical Manual of Mental Disorders.

[8] Becker N., Vasconcelos M., Oliveira V., Santos F., Bizarro L., Almeida R., Salles J.F., Carvalho M. (2017). Genetic and environmental risk factors for developmental dyslexia in children: Systematic review of the last decade. Dev. Neuropsychol..

[9] Ramus F., Altarelli I., Jednoróg K., Zhao J., Scotto di Covella L. (2018). Neruoanatomy of developmental dyslexia: Pitfalls and promise. Neurosci. Biobehav. Rev..

[10] Richlan F., Kronbichler M., Wimmer H. (2009). Functional abnormalities in the dyslexic brain: A quantitative meta-analysis of neuroimaging studies. Hum. Brain Mapp..

[11] Richlan F., Kronbichler M., Wimmer H. (2011). Meta-analyzing brain dysfunctions in dyslexic children and adults. Neuroimage.

[12] Shaywitz S.E., Shaywitz B.A., Kliegman R.M., Stanton B.F., Geme J.W., Schor N.F. (2020). Nelson Textbook of Pediatrics.

[13] Fletcher J.M. (2009). Dyslexia: The evolution of a scientific concept. J. Int. Neuropsychol. Soc..

[14] Peterson R.L., Pennington B.F. (2015). Developmental Dyslexia. Annu. Rev. Clin. Psychol..

[15] Colletti L.F. (1979). Relationship between pregnancy and birth complications and the later development of learning disabilities. J. Learn. Disabil..

[16] Hill S.K., Cawthorne V., Dean R.S. (1998). Utilitiy of the Maternal Perinatal Scale (MPS) in distinguishing normal from learning disabled children. Int. J. Neurosci..

[17] Liu L., Wang J., Shanshan S., Luo X., Kong R., Zhang X., Song R. (2016). Descriptive epidemiology of prenatal and perinatal risk factors in a Chinese population with reading disorder. Sci. Rep..

[18] Xue J.A. (1996). Perinatal Complications as Predictors of Neuropsychological Outcome in Children with Learning Disabilities. Ph.D. Thesis.

[19] Labouesse M.A., Langhans W., Meyer U. (2015). Long-term pathological consequences of prenatal infection: Beyond brain disorders. Am. J. Physiol. Regul. Integr. Comp. Psysiol..

[20] Ranasinghe S., Or G., Wang E.Y., Levins A., McLean M.A., Niell C.M., Chau V. (2015). Reduced cortical activity impairs development and plasticity after neonatal hypoxia ischemia. J. Neurosci..

[21] Rosen M.G., Debanne S.M., Thompson K., Dickinson J.C. (1992). Abnormal labor and infant brain damage. Obstet. Gynecol..

[22] Chyi L.J., Lee H.C., Hintz S.R., Gould J.B., Sutcliffe T.L. (2010). School outcomes of late preterm infants: Special needs and challenges for infants born at 32 to 36 weeks gestation. J. Pediatr..

[23] Guarini A., Sansavini A., Fabbri C., Savini S., Alessandroni R., Faldella G., Karmiloff-Smith A. (2010). Long-term effects of preterm birth on language and literacy at eight years. J. Child Lang..

[24] Hall A., McLeod A., Counsell C., Thomson L., Mutch L. (1995). School attainment, cognitive ability and motor function in a total Scottish very-low-birthweight population at eight years: A controlled study. Dev. Med. Child Neurol..

[25] Jansson-Verkasalo E., Korpilathi P., Jäntti V., Valkama M., Vainionpää L., Alku P., Suominen K., Näätänen R. (2004). Neurophysiologic correlates of deficit phonological representations and object naming in prematurely born children. Clin. Neurophysiol..

[26] Johnson S., Wolke D., Hennessy E., Marlow N. (2011). Educational outcomes in extremely preterm children: Neuropsychological correlates and predictors of attainment. Dev. Neuropsychol..

[27] Kovachy V.N., Adams J.N., Tamaresis J.S., Feldman H.M. (2015). Reading abilities in school-aged preterm children: A review and meta-analysis. Dev. Med. Child Neurol..

[28] Marlow N., Wolke D., Bracewell M., Samara M., EPICure Study Group (2005). Neurologic and developmental disability at six years of age after extremely preterm birth. N. Engl. J. Med..

[29] Morse S.B., Zheng H., Tang Y., Roth J. (2009). Early school age outcomes of late preterm infants. Pediatrics.

[30] Roberts G., Lim J., Doyle L.W., Anderson P.J. (2011). High rates of school readiness difficulties at 5 years of age in very preterm infants compared with term controls. J. Dev. Behav. Pediatr..

[31] Wolke D., Samara M., Bracewell M., Marlow N., EPICure Study Group (2008). Specific language difficulties and school achievement in children born at 25 weeks of gestation or less. J. Pediatr..

[32] Holm A., Crosbie S. (2010). Literacy skills of children born preterm. Aust. J. Learn. Diffic..

[33] Russell R.B., Petrini J.R., Damus K., Mattison D.R., Schwarz R.H. (2003). The changing epidemiology of multiple births in the United States. Obstetrics Gynecol..

[34] Cleary-Goldman J., Malone F.D., Vidaver J., Ball R.H., Nyberg D.A., Comstock C.H., Saade G.R., Eddleman K.A., Klugman S., Dugoff L. (2005). Impact of maternal age on obstetric outcome. Obstet. Gynecol..

[35] McLennan A.S., Gyamfi-Bannerman C., Ananth C.V., Wright J.D., Siddiq Z., D’Alton M.E., Friedman A.M. (2017). The role of maternal age in twin pregnancy outcomes. Am. J. Obstet. Gynecol..

[36] Bogner G., Wallner V., Fazelnia C., Strobl M., Volgger B., Fischer T., Jacobs V.R. (2018). Delivery of the second twin: Influence of presentation on neonatal outcome, a case controlled study. BMC Pregnancy Childbirth.

[37] Seelbach-Goebel B. (2014). Twin Birth Considering the Current Results of the “Twin Birth Study”. Geburtshilfe Frauenheilkd..

[38] Barrett J.F., Hannah M.E., Hutton E.K., Willan A.R., Allen A.C., Armson B.A., Gafni A., Joseph K.S., Mason D., Ohlsson A. (2013). A randomized trial of planned cesarean or vaginal delivery for twin pregnancy. N. Engl. J. Med..

[39] Pasupathy D., Smith G.C. (2008). Neonatal outcomes with caesarean delivery al term. Arch. Dis. Child Fetal Neonatal Ed..

[40] Robbins L.S., Blanchard C.T., Biasini F.J., Powell M.F., Casey B.M., Tita A.T., Harper L.M. (2020). General anesthesia for cesarean delivery and childhood neurodevelopmental and perinatal outcomes: A secondary analysis of a randomized controlled trial. Int. J. Obstet. Anesth..

[41] González-Mesa E., Cazorla-Granados O., González-Valenzuela M.J. (2016). The influence of obstetric variables on school achievement, intelligence and neuropsychological development in a sample of Spanish twins at the age of six: A retrospective study. J. Matern. Fetal Neonatal Med..

[42] González-Valenzuela M.J., González-Mesa E., Cazorla-Granados O., López-Montiel D. (2019). Type of Delivery, Neuropsychological Development and Intelligence in Twin Births. Front. Psychol..

[43] González-Valenzuela M.J., López-Montiel D., Cazorla-Granados O., González-Mesa E. (2019). Type of delivery and reading, writing, and arithmetic learning in twin births. Dev. Psychobiol..

[44] Fletcher J.M., Coulter W.A., Reschly D.J., Vaughn S. (2004). Alternative approaches to definition and identification on learning disabilities: Some questions and answers. Ann. Dyslexia..

[45] García-Vidal J., González-Manjón D., Ortiz B.G. (2011). Batería Psicopedagógica EVALÚA-1.

[46] González-Valenzuela M.J., Martín-Ruiz I. (2017). Effects on Reading of an Early Intervention Program for Spanish Children at Risk of Learning Difficulties. RASE.

[47] Welsch R.G. (2007). Using experimental analysis to determine interventions for reading fluency and recalls of students with learning disabilities. Learn. Disabil. Q..

[48] Kaufman A.S., Kaufman N.L. (2000). Test Breve de Inteligencia de Kaufman (K. BIT).

[49] Cohen J. (1998). Statistical Power Analysis for the Behavioral Sciences.

[50] Tomczak A., Tomczak E. (2014). The need to report effect size estimates revisited. An overview of some recommended measures of effect size. Trends Sport Sci..

[51] Hosmer D.W., Lemeshow S. (2000). Applied Logistic Regression.

[52] Kleinbaum D.G., Klein M. (2001). Logistic Regression: A Self-Learning Text.

[53] Chen H., Cohen P., Chen S. (2010). How big is a big odds ratio? Interpreting the magnitudes of odds ratios in epidemiological studies. Commun. Stat. Simul. Comput..

[54] Efron B., Tibshirani R.J. (1993). An Introduction to the Bootstrap.

[55] Simón-Areces J., Dietrich M., Hermes G., García-Segura L., Arévalo M., Horvath T. (2012). Ucp2 induced by natural birth regulates neuronal differentiation of the hippocampus and related adult behavior. PloS ONE.

[56] Saccone G., Berghella V. (2016). Antenatal corticosteroids for maturity of term or near term fetuses: Systematic review and meta-analysis of randomized controlled trials. BMJ.

[57] El-Khodor B.F., Boksa P. (1997). Long-term reciprocal changes in dopamine levels in prefrontal cortex versus nucleus accumbens in rats born by Caesarean section compared to vaginal birth. Exp Neurol..

[58] Vaillancourt C., Boksa P. (2000). Birth insult alters dopamine-mediated behavior in a precocial species, the guinea pig. Implications for schizophrenia. Neuropsychopharmacology.

[59] El-Khodor B.F., Boksa P. (2003). Differential vulnerability of male versus female rats to long-term effects of birth insult on brain catecholamine levels. Exp. Neurol..

[60] Van Handel M., Swaab H., de Vries L.S., Jongmans M.J. (2007). Long-term cognitive and behavioral consequences of neonatal encephalopathy following perinatal asphyxia: A review. Eur. J. Pediatr..

[61] Hokkanen L., Launes J., Michelsson K. (2014). Adult neurobehavioral outcome of hyperbilirubinemia in full term neonates-a 30-year prospective follow-up study. PeerJ.

[62] Cho K., Frijters J.C., Zhang H., Miller L.L., Gruen J.R. (2013). Prenatal exposure to nicotine and impaired reading performance. J. Pediatr..

[63] Mascheretti S., Bureau A., Battaglia M., Simone D., Quadrelli E., Croteau J., Cellino M.R., Giorda R., Beri S., Maziade M. (2013). An assessment of gene-by-environment interactions in developmental dyslexia-related phenotypes. Genes Brain Behav..

